# Supplementary material for: Coordination Behavior of 1,4-Disubstituted Cyclen Endowed with Phosphonate, Phosphonate Monoethylester, and H-Phosphinate Pendant Arms
Source: Molecules. 2019 Sep 12;24(18):3324. doi: 10.3390/molecules24183324 (PMC6767212; doi:10.3390/molecules24183324)
Supplement: Supplementary file 1 [file molecules-24-03324-s001.pdf]

Supplementary Materials

Coordination Behavior of 1,4-Disubstituted Cyclen Endowed with Phosphonate, Phosphonate Monoethylester, and H-Phosphinate Pendant Arms

Jiří Bárta, Petr Hermann and Jan Kotek *

Department of Inorganic Chemistry, Faculty of Science, Charles University, Hlavova 8, 128 43 Prague 2, Czech Republic; BJRS@seznam.cz (J.B.); petrh@natur.cuni.cz (P.H.)

* Correspondence: modrej@natur.cuni.cz

Content:

Synthesis of 1,4-dibenzyl-1,4,7,10-tetraazacyclododecane (**1**)
Figure S1. Hydrogen bond network found in the crystal structure of 1,4-H_4do2p·4H_2O.
Table S1. Geometric parameters of hydrogen bond network found in the crystal structure of 1,4-H_4do2p·4H_2O.
Table S2. Experimental values of overall protonation/stability constants (logβ_{hlm}) of the ligands 1,4-H_4do2p, 1,4-H_2do2p^{OEt} and 1,4-H_2Bn_2do2p^{H} and their complexes with metal ions.
Figure S2. Distribution diagram of differently protonated species of studied ligands.
Figure S3. Distribution diagram of species present in Cu(II)–studied ligand mixtures.
Figure S4. Distribution diagram of species present in Zn(II)–studied ligand mixtures.
Figure S5. ^{1}H NMRD profiles of Mn(II)–1,4-H_2do2p^{OEt} and Mn(II)–1,4-H_2Bn_2do2p^{H} complexes.
Figure S6. Temperature dependence of $^5D_0 \leftarrow {}^7F_0$ transition in absorption spectra of Eu(III)–1,4-H_4do2p complex.

Synthesis of 1,4-dibenzyl-1,4,7,10-tetraazacyclododecane (1)

Cyclen was obtained from Chematech and was used as received. Oxalyl-protected cyclen and its dibenzyl derivative were obtained according to ref.[1]. The 1,4-dibenzylcyclen was obtained by slightly modified literature method:[1]

Dibenzyl-oxalyl-cyclen (9.0 g, 22 mmol) was dissolved in a mixture 30% aq. NaOH (100 ml) and EtOH (20 ml). The solution was stirred and heated to 95 °C overnight. The reaction mixture was cooled and the volatiles were evaporated in vacuum. The solid was dissolved in water (70 ml) and washed by CH_2Cl_2 (100 ml) three times. The CH_2Cl_2 phases were combined, the solution was dried by anhydrous Na_2SO_4 and volatiles were evaporated in vacuum. The oily residue solidified in fridge. The white solid was dried in vacuo (7.0 g, 90 %). Spectral data were consistent with those reported in literature.[1]

[1] Bellouard, F.; Chuburu, F.; Kervarec, N.; Toupet, L.; Triki, S.; Le Mest, Y.; Handel, H. *cis*-Diprotected cyclams and cyclens: a new route to symmetrically or asymmetrically 1,4-disubstituted tetraazamacrocycles and to asymmetrically tetrasubstituted derivatives. *J. Chem. Soc., Perkin Trans. 1* **1999**, 3499–3505.

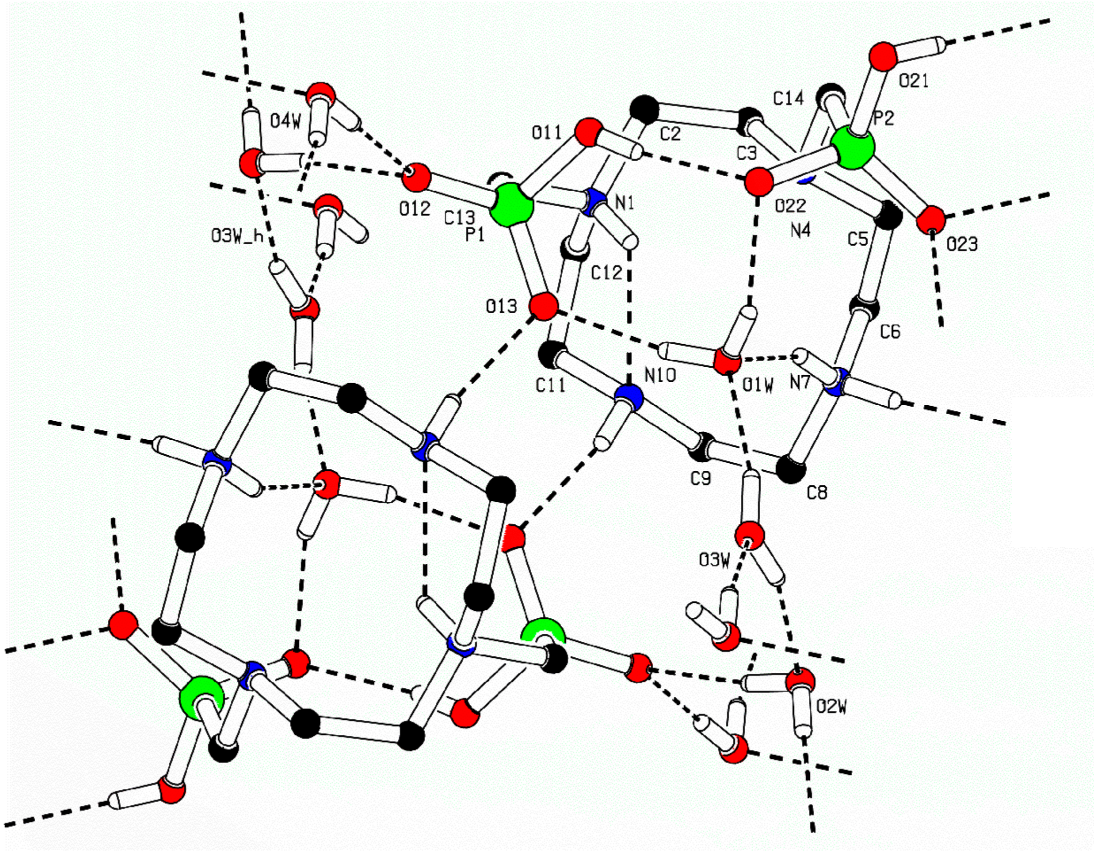

Figure S1. Hydrogen bond network found in the crystal structure of 1,4-$H_4do2p \cdot 4H_2O$. Carbon-bound hydrogen atoms are omitted for clarity.

Table S1. Geometric parameters of hydrogen bond network found in the crystal structure of 1,4-H_4do2p·4H_2O.

D–H	d(D–H) [Å]	d(H···A) [Å]	<D-H···A [°]	d(D···A) [Å]	A
N1-H11	0.86(3)	2.64(3)	110(2)	3.040(1)	N4
N1-H11	0.86(3)	2.30(3)	118(2)	2.808(2)	N10
N7-H71	0.88(2)	2.65(5)	107(1)	3.030(2)	N4
N7-H71	0.88(2)	2.75(2)	97(1)	2.989(2)	N10
N7-H71	0.88(2)	2.03(2)	153(2)	2.847(2)	O1W
N7-H72	0.91(2)	1.82(2)	162(2)	2.704(2)	O4W [*x*−1, *y*−1, *z*]
N10-H101	0.88(2)	1.99(2)	161(2)	2.839(2)	O13 [−*x*+1, −*y*+1, −*z*+1]
O11-H11O	0.72(2)	1.89(2)	170(3)	2.603(1)	O22
O21-H221	0.78(2)	1.83(2)	178(2)	2.608(1)	O23 [−*x*+1, −*y*, −*z*]
O1W-H11W	0.86(2)	1.83(3)	168(2)	2.684(2)	O13
O1W-H12W	0.83(2)	1.99(2)	160(2)	2.785(1)	O22
O2W-H21W	0.86(2)	1.92(2)	172(2)	2.779(2)	O12 [−*x*+1, −*y*+1, −*z*+1]
O2W-H22W	0.83(2)	2.00(2)	178(2)	2.825(1)	O23 [−*x*+1, −*y*, −*z*+1]
O3W-H31W	0.87(3)	1.92(3)	164(2)	2.772(2)	O2W
O3W-H32W	0.87(3)	1.90(3)	166(2)	2.748(2)	O1W
O4W-H41W	0.87(3)	1.81(3)	165(2)	2.666(2)	O3W [−*x*+2, −*y*+1, −*z*+1]
O4W-H42W	0.82(2)	1.89(2)	174(2)	2.704(2)	O12

Table S2. Experimental values of overall protonation/stability constants (logβ_{hlm})[a] of 1,4-H_4do2p, 1,4-H_2do2p^{OEt} and 1,4-H_2Bn_2do2p^H and their complexes with metal ions. Conditions: 25 °C, I = 0.1 M $(NMe_4)Cl$. Standard deviations in parenthesis as calculated by OPIUM.

Ion	Equilibrium[b]	*h*	*l*	*m*	1,4-H_4do2p	1,4-H_2do2p^{OEt}	1,4-H_2Bn_2do2p^H
H^+	$\mathbf{L}^{n-} + H^+ \rightleftharpoons H\mathbf{L}^{1-n}$	0	1	0	12.84(2)	11.377(6)	11.249(9)
	$\mathbf{L}^{n-} + 2\,H^+ \rightleftharpoons H_2\mathbf{L}^{2-n}$	1	1	0	24.04(2)	19.95(1)	18.08(2)
	$\mathbf{L}^{n-} + 3\,H^+ \rightleftharpoons H_3\mathbf{L}^{3-n}$	2	1	0	31.79(2)	21.24(2)	–
	$\mathbf{L}^{n-} + 4\,H^+ \rightleftharpoons H_4\mathbf{L}^{4-n}$	3	1	0	36.73(2)	–	–
	$\mathbf{L}^{n-} + 5\,H^+ \rightleftharpoons H_5\mathbf{L}^{5-n}$	4	1	0	38.13(2)	–	–
Mg^{2+}	$\mathbf{L}^{n-} + Mg^{2+} \rightleftharpoons [Mg(\mathbf{L})]^{2-n}$	0	1	0	7.36(1)	–	–
	$\mathbf{L}^{n-} + Mg^{2+} + H^+ \rightleftharpoons [Mg(H\mathbf{L})]^{3-n}$	1	1	0	17.07(2)	–	–
	$\mathbf{L}^{n-} + Mg^{2+} + 2\,H^+ \rightleftharpoons [Mg(H_2\mathbf{L})]^{4-n}$	2	1	0	26.31(3)	–	–
Ca^{2+}	$\mathbf{L}^{n-} + Ca^{2+} \rightleftharpoons [Ca(\mathbf{L})]^{2-n}$	0	1	1	9.29(2)	5.90(2)	3.96(3)
	$\mathbf{L}^{n-} + Ca^{2+} + H^+ \rightleftharpoons [Ca(H\mathbf{L})]^{3-n}$	1	1	1	18.31(2)	–	–
	$\mathbf{L}^{n-} + Ca^{2+} + H_2O \rightleftharpoons [Ca(OH)(\mathbf{L})]^{1-n} + H^+$	−1	1	1	–	–	−8.32(4)
Mn^{2+}	$\mathbf{L}^{n-} + Mn^{2+} \rightleftharpoons [Mn(\mathbf{L})]^{2-n}$	0	1	1	15.41(1)	11.420(9)	9.39(2)
	$\mathbf{L}^{n-} + Mn^{2+} + H^+ \rightleftharpoons [Mn(H\mathbf{L})]^{3-n}$	1	1	1	23.513(4)	–	–
	$\mathbf{L}^{n-} + Mn^{2+} + 2\,H^+ \rightleftharpoons [Mn(H_2\mathbf{L})]^{4-n}$	2	1	1	28.74(2)	–	–
	$\mathbf{L}^{n-} + Mn^{2+} + H_2O \rightleftharpoons [Mn(OH)(\mathbf{L})]^{1-n} + H^+$	−1	1	1	–	−0.78(2)	−1.74(2)
Cu^{2+}	$\mathbf{L}^{n-} + Cu^{2+} \rightleftharpoons [Cu(\mathbf{L})]^{2-n}$	0	1	1	26.45(4)	19.95(3)	–
	$\mathbf{L}^{n-} + Cu^{2+} + H^+ \rightleftharpoons [Cu(H\mathbf{L})]^{3-n}$	1	1	1	33.13(4)	–	–
	$\mathbf{L}^{n-} + Cu^{2+} + 2\,H^+ \rightleftharpoons [Cu(H_2\mathbf{L})]^{4-n}$	2	1	1	37.80(3)	–	–
	$\mathbf{L}^{n-} + Cu^{2+} + H_2O \rightleftharpoons [Cu(OH)(\mathbf{L})]^{1-n} + H^+$	−1	1	1	–	7.24(7)	–
Zn^{2+}	$\mathbf{L}^{n-} + Zn^{2+} \rightleftharpoons [Zn(\mathbf{L})]^{2-n}$	0	1	1	21.111(9)	15.416(6)	13.26(2)
	$\mathbf{L}^{n-} + Zn^{2+} + H^+ \rightleftharpoons [Zn(H\mathbf{L})]^{3-n}$	1	1	1	27.998(5)	–	–
	$\mathbf{L}^{n-} + Zn^{2+} + 2\,H^+ \rightleftharpoons [Zn(H_2\mathbf{L})]^{4-n}$	2	1	1	32.110(6)	–	–
	$\mathbf{L}^{n-} + Zn^{2+} + H_2O \rightleftharpoons [Zn(OH)(\mathbf{L})]^{1-n} + H^+$	−1	1	1	8.30(2)	5.91(1)	4.14(4)
	$\mathbf{L}^{n-} + Zn^{2+} + 2\,H_2O \rightleftharpoons [Zn(OH)_2(\mathbf{L})]^{-n} + 2\,H^+$	−2	1	1	–	–	−8.17(5)
Gd^{3+}	$\mathbf{L}^{n-} + Gd^{3+} \rightleftharpoons [Gd(\mathbf{L})]^{3-n}$	0	1	1	19.15(5)	–	–
	$\mathbf{L}^{n-} + Gd^{3+} + H^+ \rightleftharpoons [Gd(H\mathbf{L})]^{4-n}$	1	1	1	26.20(5)	–	–
	$\mathbf{L}^{n-} + Gd^{3+} + 2\,H^+ \rightleftharpoons [Gd(H_2\mathbf{L})]^{5-n}$	2	1	1	32.22(3)	–	–
	$\mathbf{L}^{n-} + Gd^{3+} + 3\,H^+ \rightleftharpoons [Gd(H_3\mathbf{L})]^{6-n}$	3	1	1	36.08(4)	–	–

[a] $\beta_{hlm} = [H_hL_lM_m]/([H]^h\cdot[L]^l\cdot[M]^m)$. [b] n = 4 for 1,4-H_4do2p, and n = 2 for 1,4-H_2do2p^{OEt} and 1,4-H_2Bn_2do2p^H.

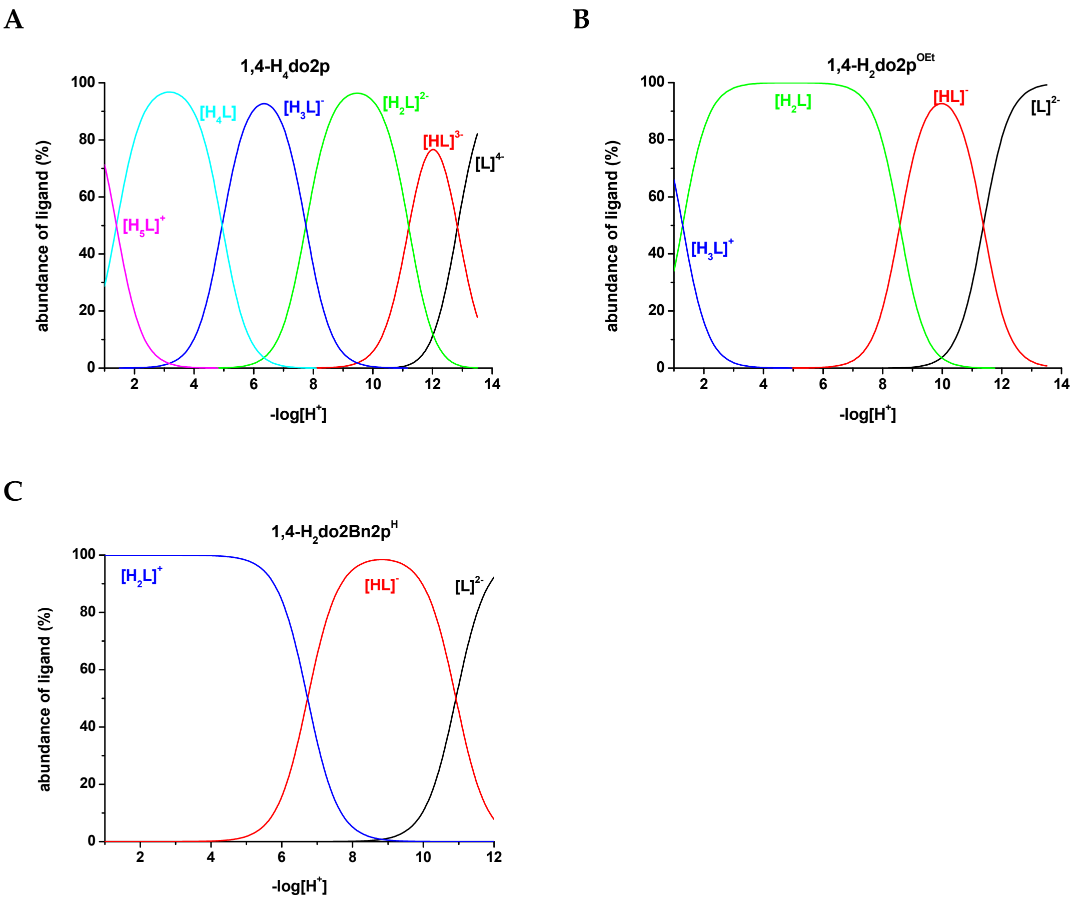

Figure S2. Distribution diagram of differently protonated species of **A**: 1,4-H_4do2p; **B**: 1,4-H_2do2p^{OEt}; and **C**: 1,4-H_2Bn_2do2p^{H} (c_L = 5 mM, I = 0.1 M NMe_4Cl, 25 °C).

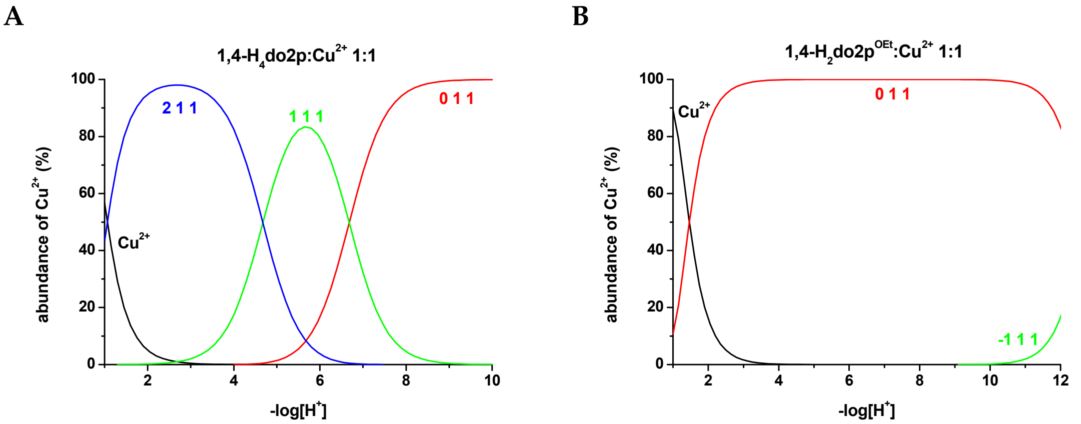

Figure S3. Distribution diagram of species present in (**A**): Cu(II)–1,4-H_4do2p system; and (**B**): Cu(II)–1,4-H_2do2p^{OEt} system. Stoichiometric coefficients: H-L-M.

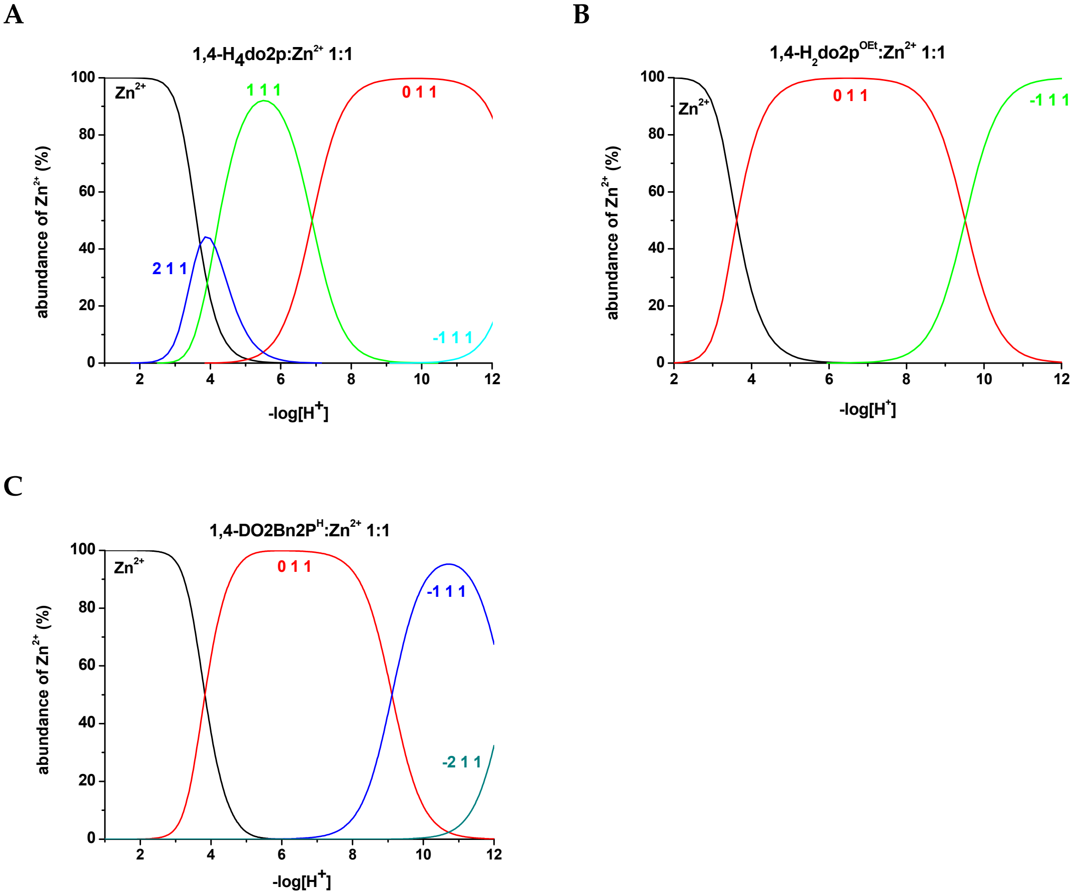

Figure S4. Distribution diagram of species present in (**A**): Zn(II)–1,4-H_4do2p system; (**B**): Zn(II)–1,4-H_2do2p^{OEt} system; and (**C**): Zn(II)–1,4-H_2Bn_2do2p^{H} system ($c_L = c_{Zn}$ = 5 mM, I = 0.1 M NMe_4Cl, 25 °C). Stoichiometric coefficients: H-L-M.

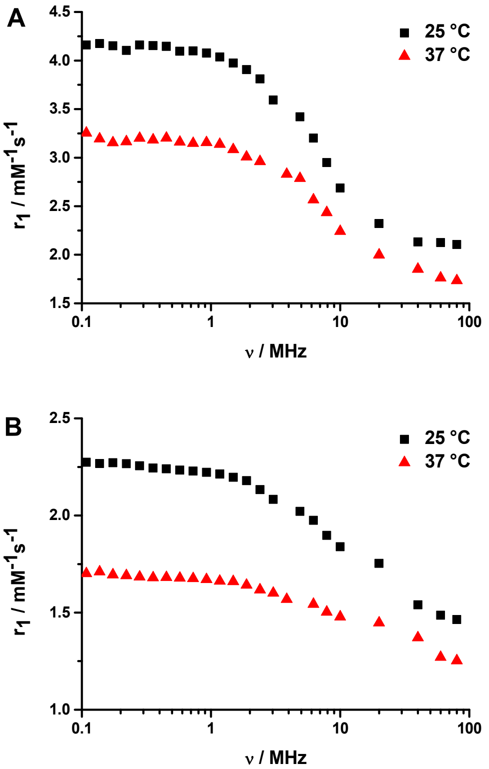

Figure S5. ^{1}H NMRD profiles of **A**: Mn(II)–1,4-H_2do2p^{OEt} (pH 9.0, ~100% [Mn(1,4-do2p^{OEt})]); and **B**: Mn(II)–1,4-$H_2$$Bn_2$do2p^H (pH 9.0, ~100% [Mn(Bn_2do2p^H)]) systems.

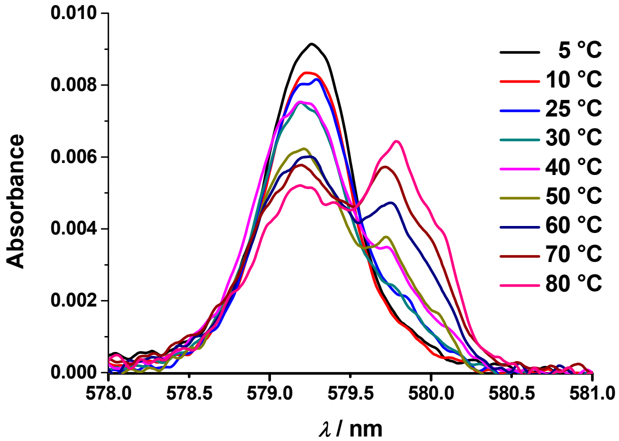

Figure S6. Temperature dependence of $^5D_0 \leftarrow {^7F_0}$ transition in absorption spectrum of Eu(III)–1,4-H_4do2p complex.
